# Dark Sweet Cherry (*Prunus avium* L.) Juice Phenolics Rich in Anthocyanins Exhibit Potential to Inhibit Drug Resistance Mechanisms in 4T1 Breast Cancer Cells via the Drug Metabolism Pathway

**DOI:** 10.3390/cimb47030213

**Published:** 2025-03-20

**Authors:** Ana Nava-Ochoa, Susanne U. Mertens-Talcott, Stephen T. Talcott, Giuliana D. Noratto

**Affiliations:** Department of Food Science and Technology, Texas A&M University, College Station, TX 77843, USA; ana.nava@tamu.edu (A.N.-O.); smtalcott@tamu.edu (S.U.M.-T.); stalcott@tamu.edu (S.T.T.)

**Keywords:** anthocyanins, dark sweet cherries, polyphenols, breast cancer, drug metabolism, drug resistance, chemoresistance

## Abstract

Anthocyanins (ACNs) from dark sweet cherries (DSCs) have shown efficacy against breast cancer (BC) cells, particularly triple-negative breast cancer (TNBC) cells, without affecting normal breast cells. This study investigated the impact of ACNs on TNBC cells, focusing on drug resistance mechanisms involving drug metabolism and transport enzymes. Specifically, it was examined whether ACNs influenced Doxorubicin (DOX) metabolism by targeting drug metabolism enzymes (phase I metabolism) and drug transport enzymes (phase III metabolism) in TNBC cells. 4T1 TNBC cells were treated with ACNs, DOX, and the combination of both (ACN-DOX). Results showed a synergistic inhibition of cell viability by ACNs and DOX. In addition, the modulation of phase I drug-metabolizing enzymes was exerted by ACNs, reducing the activity of cytochrome P450 (CYP) enzymes induced by DOX. A reduction of drug efflux by ACNs was shown by decreasing P-glycoprotein (P-gp) activity, leading to a higher intracellular accumulation of DOX. These effects were confirmed using CYP and P-gp inducers and inhibitors, showing their impact on cell viability. In conclusion, the combination of ACNs with DOX has the potential to lower DOX doses, enhance its efficacy, and possibly reduce side effects, offering a promising approach for TNBC treatment.

## 1. Introduction

Triple negative breast cancer (TNBC) refers to cancer cells that lack expression of the human epidermal growth factor receptor type 2 (HER2), estrogen receptor (ER), and progesterone receptor (PR), and therefore are unable to be treated with endocrine therapy or receptor-targeted drug therapies, which are commonly used treatments for breast cancer (BC) [1]. As one of the most aggressive forms of BC, TNBC cells have been shown to have heightened chemoresistance, a common effect when implementing conventional cancer treatments.

Doxorubicin (DOX) is a commonly used and effective chemotherapeutic drug for treating TNBC. Despite its reported effectiveness, cells can develop mechanisms to protect themselves against its effects. These mechanisms include decreased drug uptake, increased drug efflux, and enhanced drug inactivation through biotransformation [2]. DOX also shows undesired effects in healthy cells, mainly leading to cardiotoxicity, hepatoxicity, and nephrotoxicity [3]. Combination therapies with DOX have been reported as alternatives to reduce its side effects. Although some studies have shown the potential of these combinations, they have also been shown to develop cytotoxicity and drug resistance over time [4]. Studying the co-administration of DOX with other compounds is appealing as it may help identify those that could reduce its side effects.

Dark sweet cherries (*Prunus avium* L.) (DSCs) are high in phenolic compounds which have shown anticarcinogenic properties. In previous studies, DSC juice phenolics enriched in anthocyanins (ACNs) have shown activity against the growth of the most aggressive BC subtypes without toxicity in normal breast cells [5]. Previous studies have shown that these compounds can have an effect in the drug metabolism process, targeting phase I enzymes responsible of xenobiotic metabolism and phase III transporters responsible for active transport of drugs out of the cell [6,7,8]. The upregulation of these proteins in cancer cells is believed to be linked with drug resistance (DR) [9].

Phase I enzymes are part of the cytochrome P450 (CYP) family, which are key targets in cancer prevention. These enzymes play a crucial role in xenobiotic metabolism [10]. Studies have reported the presence of CYP1 family enzymes, specifically CYP1A1 and CYP1B1, in mammary tissue and their overexpression in tumor tissue and cancer cells [11,12]. These two enzymes are transcriptionally regulated by the aryl hydrocarbon receptor (AhR), which is activated by the presence of exogenous compounds. Their high expression is sometimes correlated with a negative breast cancer prognosis and a contribution to carcinogenesis [11,13].

Phase III drug transporters are known to contribute to drug resistance (DR), primarily due to their ability to increase drug efflux from the cell, thereby lowering the intracellular concentration of drugs. Most of these transporters belong to the ATP-Binding Cassette (ABC) superfamily, which uses energy from ATP to pump drugs out of the cell. Among these transporters, P-glycoprotein (P-gp), also known as multidrug resistance protein 1 (MDR1) or ATP-Binding Cassette Subfamily Member 1 (ABCB1), the breast cancer resistance protein (BCRP), also known as ATP-Binding Cassette Subfamily G Member 2 (ABCG2), and the multidrug resistance protein 2 (MRP2), also known as ATP-Binding Cassette Subfamily C Member 2 (ABCC2), are the most commonly overexpressed transporters in TNBC that contribute to DR [14,15]. The role of these proteins has been of research interest as a therapeutic treatment to lower or prevent drug resistance.

The 4T1 cell line is commonly used as a model for studying stage IV human BC due to its high metastatic potential, allowing it to spread from the primary tumor in the mammary gland to distant organs such as the lungs, liver, and bones. Previous studies using DSCs have demonstrated the effectiveness of ACNs in modulating markers downstream of the MAPK signaling pathway [16] which are commonly targeted to treat TNBC cells. As for DR targets, the effect of ACNs has not been reported but the effect of other polyphenols has been widely studied [13,16,17,18].

The modulation of CYPs by plant-based products has been studied in several types of cancer, including BC, and has been reported as a potential strategy to inhibit tumor growth and metastasis, partly due to their involvement in various cell signaling pathways [19]. For ABC transporters, the use of polyphenols has shown an increase in intracellular substrate accumulation related to P-gp targeting, as demonstrated in drug-resistant CHC5 ovarian cancer cells treated with green tea polyphenols [6], KB/VIN human cervical cells treated with caffeic acid [20], and Caco-2 colon cancer cells treated with curcumin [21]. The effect of polyphenols in combination with chemotherapeutic drugs has been documented in several studies [22,23,24], highlighting the relevance of studying the use of ACN in combination with the chemotherapeutic drug DOX against 4T1 BC cells.

This study hypothesizes that ACN synergizes with DOX to inhibit 4T1 cell growth. We aimed to determine whether this effect involves phase I drug-metabolizing enzymes (CYP1A1 and CYP1B1) and key phase III drug transporters (MDR-1, BCRP, and MRP2).

## 2. Materials and Methods

### 2.1. Chemicals, Antibodies, and Reagents

Solvents used to prepare the ACN extract were purchased from Fisher Chemical (Pittsburgh, PA, USA). Rhodamine-123 (Rh) was purchased from Sigma Aldrich (San Luis, MO, USA). Verapamil hydrochloride (VER) 99% was purchased from Enzo Life Sciences (Farmingdale, NY, USA). Hanks Balanced Salt Solution (HBSS), alpha-naphthoflavone (ANF), and the Halt™ Protease Inhibitor Cocktail (100X) were purchased from ThermoFisher Scientific (Grand Island, NY, USA). The xTractor™ buffer was purchased from Takara Bio Company (Mountain View, CA, USA). Antibodies for CYP1A1, CYP1B1, P-gp/MDR-1, MRP2, and BCRP as well as secondary antibodies (mouse and rabbit) were purchased from Cell Signaling Technology Inc. (Danvers, MA, USA). Nicotinamide adenine dinucleotide phosphate (NADPH) tetrasodium salt was purchased from VWR (Radnor, PA, USA). Benzo(a)pyrene (BaP) was purchased from TCI America (Portland, OR, USA).

### 2.2. Extraction of Phenolics Enriched in Anthocyanins (ACN)

Concentrated DSC juice was kindly supplied by FruitSmart Inc. (Grandview, WA, USA). Its phenolic profile was previously reported [5]. Fractionation of phenolic compounds and extraction of a phenolic fraction rich in anthocyanins (ACN) was performed as previously reported [16]. Anthocyanins were quantified by the pH-differential spectrophotometric method [25] as cyanidin-3-O-rutinoside equivalents (C3R), which is the most abundant anthocyanin in the ACN rich fraction. C3R accounts for 79% of the total anthocyanins in concentrated DSC juice, followed by cyanidin-3-O-glucoside and peonidin-3-O-rutinoside, which account for 9% and 8% of total anthocyanins, respectively [5]. Extraction and quantification of ACNs were performed before each experiment to ensure the standardization of the ACN extract.

### 2.3. Cell Line

Derived from mouse mammary glands, 4T1 cells mimic stage IV human BC. The TNBC 4T1 cells were purchased from ATCC (Manassas, VA, USA). The 4T1 cells were grown in RPMI-1640 culture medium purchased from ATCC (catalog #30-2001) supplemented with 10% (*v*/*v*) fetal bovine serum (FBS) (Gibco, Invitrogen Corp., Grand Island, NY, USA) and 1% (*v*/*v*) penicillin–streptomycin antibiotic mix (P/S) (ThermoFisher Scientific, Grand Island, NY, USA). The cells were maintained at 37 °C with a humidified 5% CO_2_ atmosphere, as recommended by ATCC.

### 2.4. Cell Viability

The resazurin in vitro toxicology assay kit (Sigma-Aldrich, St. Louis, MO, USA) was selected for cell viability testing due to its low variability within and between plates. The Resazurin assay was performed according to the manufacturer’s protocol. Briefly, cells seeded in 96-well plates were allowed to reach ~75% confluence, followed by treatment with ACN (0–300 μg C3R/mL) and/or DOX (0–15 μg/mL) for 48 h. Relative fluorescence units (RFUs) were measured at 560 and 590 nm excitation and emission wavelengths, respectively, using the Clariostar plate reader (BMG Labtech Inc., Durhan, NC, USA). Cell viability (% of control) from *n* ≥ 3 was calculated as the percentage of DMSO-treated control cells (control). The dose needed to inhibit cell viability by 50% (IC_50_) was calculated from at least three independent determinations. Controls were treated with up to 0.2% *v*/*v* dimethyl sulfoxide (DMSO), used as a solvent of ACN and DOX.

### 2.5. Synergistic Effect of ACN and DOX Combination

Cell viability was determined using the resazurin in vitro toxicology assay kit after a 48 h treatment with ACN and DOX combinations at 0.25×, 0.5×, 1×, or 1.5× their IC_50_ (Table 1). 

The combination index (CI) was determined using the Chou–Talalay method with the CompuSyn application (www.combosyn.com, accessed on 12 June 2023). This method determines quantitatively the effect of drug combinations by inputting the doses and effects of two or more drugs, both separately and in combination, into the software [26]. The CI values of ACN and DOX indicate a strong synergism for values of CI < 0.3, synergism for values 0.3 ≤ CI < 0.7, moderate synergism for values 0.70 ≤ CI < 0.85, slight synergism for values 0.85 ≤ CI < 1, additive for CI = 1, and antagonist for CI > 1, as determined by the program’s user’s guide. The combination exerting synergism (ACN-DOX) was used for further experiments.

### 2.6. Gene Expression

Cells seeded in 6-well plates were allowed to reach ~85–90% confluence, followed by treatment with ACN (IC_50_), DOX (IC_50_) and ACN-DOX in culture medium supplemented with 2.5% FBS. Gene expression levels were determined after a 6 h treatment. The incubation period was selected based on the observed maximum modulation of the target genes of interest within a 2–24 h timeframe. The mRNA extraction was performed using the Zymo Quick-RNA™ Microprep Kit (Zymo Research, Irvine, CA, USA) following the manufacturer’s protocol. The reverse transcription Supermix iScriptTM (BioRad Laboratories, Hercules, CA, USA) was used for cDNA synthesis followed by real-time polymerase chain reaction (RT-PCR) amplification using SsoAdvancedTM Universal SYBR^®^ Green Supermix (BioRad Laboratories, Hercules, CA, USA). Relative mRNA levels were calculated using Ribosomal Protein L19 (RPL-19) as a housekeeping gene, employing the comparative CT method as described [27]. Mouse primers for CYP1A1 and CYP1B1 were purchased from Sigma Aldrich (San Louis, MO, USA). Primers for ABCB1, ABCC2, ABCG2, and RPL-19 were ordered from Integrated DNA Technologies (Coralville, IA, USA). Primers were previously validated using positive and negative controls to assess their specificity and efficiency. Primer sequences can be found in Table 2.

### 2.7. Protein Expression

Cells seeded onto 10 cm culture plates were allowed to reach ~85% confluence and starved overnight in FBS-free medium, followed by treatment with ACN, DOX, and ACN-DOX in culture medium supplemented with 2.5% FBS for 48 h. Cells were lysed using xTractor buffer supplemented with the Halt™ Protease Inhibitor Cocktail, following the manufacturer’s protocol. Mechanical cell disruption was performed by a series of steps: freezing, thawing, and sonication for 30 s. Cell lysates were subjected to Western blot analysis as reported [5] using 35 μg of protein quantified with the Bradford protein assay (BioRad Laboratories, Hercules, CA, USA). Protein bands were visualized with luminal reagent from Santa Cruz Biotechnology, Inc. (Santa Cruz, CA, USA) after 1–5 min of reaction. Band intensities were quantified using ImageJ Software in browser (https://imagej.net/ij/, accessed on 14 October 2023).

### 2.8. CYP1 Enzymatic Activity

Ethoxyresorufin-O-deethylase (EROD) assay was performed as specified [28] with slight modifications. Briefly, cells seeded in a 96-well plate were allowed to reach ~95% confluence and incubated with HBSS for 30 min followed by ACN (IC_50_), DOX (IC_50_), or ACN-DOX treatments in HBSS for 2 h. DMSO-treated cells were used as controls. Cells were washed twice with warm PBS and EROD 2 µM (VWR, Radnor, PA, USA) was added to start the reaction. The fluorescence generated by the conversion of EROD to resorufin was measured every 2 min at 535 and 585 nm excitation and emission, respectively, for 30 min at 37 °C using ClarioStar plate reader (BMG Labtech, Ortenberg, Germany). The generation of resorufin was quantified against a standard curve of resorufin (0–30 nmol). Enzymatic activity was quantified by identifying the linear portion of the generated curve and the concentrations of resorufin at the highest Relative Fluorescent Unit (RFU) and lowest RFU points were calculated against the standard curve. The enzymatic activity was calculated by the following equation: Enzymatic activity (U/mL) = $\frac{resorufin\;(∆nmoles)}{time\;(min)}/mL$. After determining enzymatic activity, the plates were rinsed twice with phosphate buffer solution (PBS). Then, 50 µL of water was added to each well to lyse the cells. The plates were placed at −80 °C for 30 min. After thawing, the Bradford protein assay was used to measure the protein concentration in each well, using a bovine serum albumin (BSA) standard curve (0–2 µg) as a reference. Enzymatic activity was then standardized to the protein amount in each well. The results represent U/mg protein, which accounts for the amount of enzyme that converts one nmol of substrate per minute. Results are presented as fold of control.

### 2.9. Effect of CYP Modulation on Cell Viability

Cells were seeded in 96-well plates and allowed to reach ~75% confluence. Cells were pre-treated with CYP inhibitor ANF (0.1 µM) or CYP inducer BaP (30 µM) for 2 h. After pre-treatment, cells were treated with ACN (0–300 µg C3R/mL) or DOX (0–15 µg/mL), maintaining ANF and BaP concentrations, and incubated for 48 h. Cell viability was determined as detailed in Section 2.4.

### 2.10. Rhodamine123 Efflux Assay

The cellular Rh efflux assay was performed as reported [20] with slight modifications. Briefly, the cells were seeded until ~95% confluence and incubated with HBSS for 30 min. After incubation, HBSS was replaced by 1 µM Rh solution in HBSS containing ACN (IC_50_), DOX (IC_50_), or the synergistic combination ACN-DOX. A positive control known to inhibit Rh efflux, VER at 25 µM, or DMSO solvent were used as controls. After incubation at 37 °C for 30 min, cells were washed twice with PBS pH 7.0 and incubated again in HBSS for 10 min at 37 °C in shaker at 200 rpm to allow fluorescent Rh efflux. The supernatant was transferred to a black 96-well plate and the RFUs of the Rh efflux were quantified at 485 nm excitation and 535nm emission, using the ClarioStar plate reader. The concentration of the Rh efflux was quantified against a Rh standard curve (0.03–1 µM) in HBSS. Intracellular Rh accumulation was calculated as fold of the VER positive control. Results were presented as mean (*n* ≥ 6) ± SD.

### 2.11. Effect of P-gp Modulation on Cell Viability

Cells were seeded in 96-well plates and allowed to reach ~75% confluence. Cells were pre-treated with P-gp inhibitor (VER, 25 µM) or P-gp inducer (BaP, 1.5 µM) for 2 h, followed by ACN (0–300 µg C3R/mL) or DOX (0–15 µg/mL) treatments, maintaining VER and BaP concentrations, for 48 h. Cell viability was determined after a 48 h incubation, following the procedure detailed in Section 2.4.

### 2.12. Statistical Analysis

Quantitative data are presented as mean values with the respective standard deviation (SD) or standard error of the mean (SE) from 3 or more replicates. Significant difference from controls was determined by one-way analysis of variance (ANOVA). Tukey’s multiple comparisons test was performed in all tests using GraphPad Prism 10 (Systat Software, Inc. San Diego CA, USA).

## 3. Results and Discussion

### 3.1. ACN and DOX Synergized to Inhibit 4T1 Cell Viability

**Figure 1:**
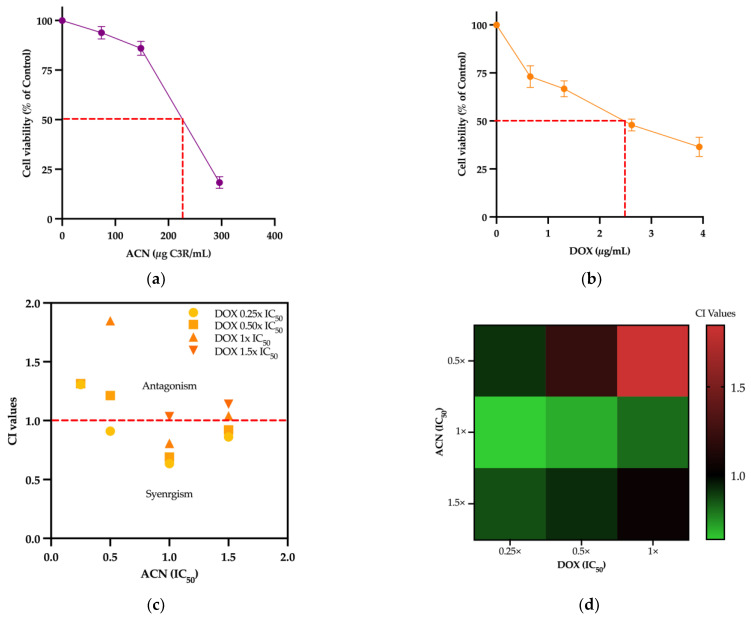
ACN inhibited cell growth and showed synergistic effects with DOX. Cell net growth of 4T1 triple negative breast cancer treated for 48 h with (**a**) ACN (0–300 µg C3R/mL) or (**b**) DOX (0–20 µg/mL). (**c**) CI values of ACN in combination with DOX. ACN IC_50_ and DOX 0.25 IC_50_ (ACN-DOX) showed the lowest CI value, having a higher synergistic effect in cell viability. (**d**) Heatmap of synergistic combinations of ACN and DOX IC_50_ proportions. Cell viability (% of DMSO control) were determined as outlined in Section 2. Values are average (*n* ≥ 3) ± SD. Synergistic combination was assessed using CompuSyn software (www.combosyn.com, accessed on 12 June 2023).

The 4T1 cell viability was inhibited in a dose-dependent manner by ACN and DOX (Figure 1a,b).

The IC_50_ for ACN and DOX were determined as 244.60 ± 31.62 µg C3R/mL and 2.51 ± 0.67 µg/mL, respectively (Table 3). 

These results confirm the antiproliferative activity of ACN against the most aggressive BC cells, as previously demonstrated [5,16,29,30].

Cell viability data from treatments with various ACN and DOX combinations (Table 2) were analyzed using the CompuSyn application (www.combosyn.com, accessed on 12 June 2023) to generate the CI values presented in Table 4. 

A synergistic activity in cell viability inhibition was identified for combinations with CI < 1, for which a greater than expected additive effect was achieved in cell viability inhibition. The combination using ACN IC_50_ and DOX 0.25× IC_50_ showed the highest synergistic effect with CI = 0.6353 and therefore was selected for future experiments aiming to investigate the underlying mechanisms (Figure 1c). Additionally, it was shown that ACN IC_50_ showed synergistic effects with all DOX IC_50_ proportions (Figure 1d).

The synergism between chemotherapeutic drugs and phenolic compounds has been widely reported [31]. Treatment of the MDA-MB-231 TNBC cell line with DOX at 0.79 µM combined with a Goji berry extract that contained flavonoids, phenolic acids, and anthocyanins, among other compounds, showed a synergistic effect with a value of CI = 0.72 [32]. In another study, it was shown that anthocyanins synergized with trastuzumab in decreasing cell growth and improved the drug apoptotic effect of HER2-positive BC cell lines MDA-MB-453, BT474, and HCC1569 [33]. Furthermore, the effect of anthocyanins from garden blueberries was evaluated in several cancer cell lines, including the MCF-7 BC cell line. Results showed enhanced antiproliferative activity values when combining anthocyanins and DOX in comparison to individual treatments [34]. Other combinations of anthocyanins with chemotherapeutic drugs have also been reviewed [23].

Among the underlying molecular mechanisms linked to the synergistic antiproliferative activity exerted by drug–phenolic combinations, the modulation of phase I drug-metabolizing enzymes and phase III drug transporters has been reported [22,35,36].

### 3.2. Modulation of Phase I Drug Metabolizing in TNBC 4T1 Cells by ACN

The CYP isoenzymes (CYPs), specifically the CYP1-4 families, play a role in metabolizing xenobiotics and cancer drugs [19]. CYPs are responsible for xenobiotic metabolism by catalyzing N-oxygenation, N-dealkylation, sulfoxidation, and formation of dehalogenated metabolites [37]. At certain conditions, active CYPs can promote drug resistance by causing the inactivation of chemotherapeutic drugs [38].

**Figure 2:**
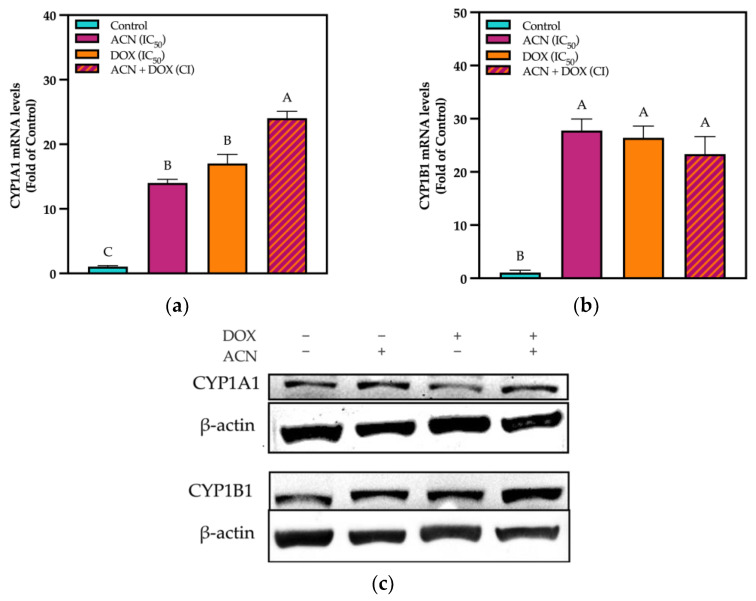
CYPs were upregulated by all treatments at gene expression while no difference was found at protein level. (**a**) CYP1A1 and (**b**) CYP1B1 mRNA levels. Cells were treated with DMSO or ACN (IC_50_), DOX (IC_50_), and ACN-DOX (CI) for 6 h. mRNA analysis was performed as outlined in Section 2. Values are average (*n* = 3) ± SEM, *p* < 0.05 compared to controls. (**c**) Effect of ACN, DOX, and ACN-DOX on expression of CYP1A1 and CYP1B1 proteins. Cell lysates were obtained after 24 h treatment with DMSO or ACN (IC_50_), DOX (IC_50_), and ACN-DOX and subjected to Western blot analysis using ß-actin as a loading control. Different capital letters indicate statistically significant differences (*p* < 0.05).

Results showed that CYP1A1 mRNA was upregulated by ACN, DOX, and ACN-DOX up to 14-, 17-, and 24-fold of the control, respectively (Figure 2a).

Likewise, CYP1B1 mRNA was upregulated by ACN, DOX, and ACN-DOX up to 28-, 26-, and 23- fold of the control, respectively (Figure 2b). However, both CYP1A1 and CYP1B1 did not show this upregulation at the protein level. No difference was found in band intensity for these two proteins (Figure 2c). The lack of correlation between CYP gene expression and protein expression has been documented. It has been hypothesized that although some flavonoids can increase CYP gene expression, this induction has shown to be further inhibited, possibly by the compounds blocking the binding of AhR to xenobiotic response elements (XRE), thus acting as inhibitors of further activity [39,40,41]. Therefore, CYP1 enzymatic activity was evaluated to determine if these compounds act as inhibitors, potentially explaining the lack of correlation between mRNA and protein expression.

**Figure 3:**
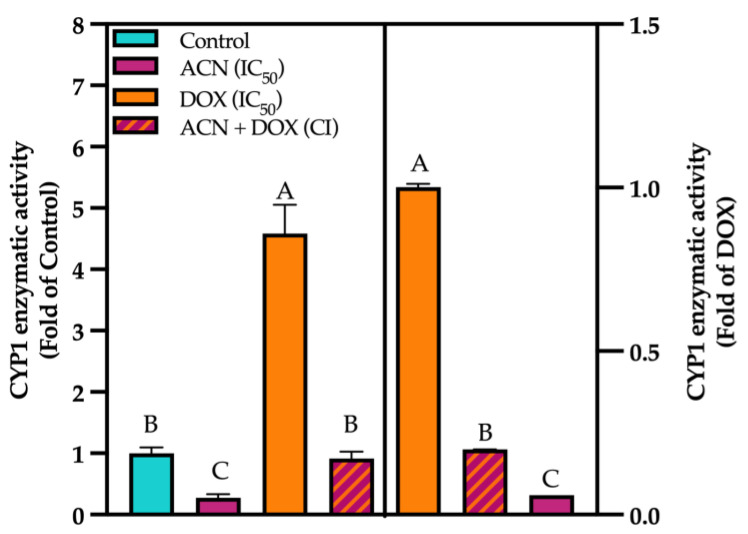
CYP activity was lowered by ACN. Enzymatic activity of CYPs. Cells were treated with DMSO or ACN (IC_50_), DOX (IC_50_), and ACN-DOX (CI) for 2 h. Resorufin production was quantified using EROD method as outlined in Section 2. Activity was compared against DMSO control (left panel) and against DOX as a control (right panel). Values are average (*n* = 3) ± SEM, *p* < 0.05. Different capital letters indicate statistically significant differences (*p* < 0.05).

Results from enzyme activity showed that DOX induced CYP1 enzymatic activity up to 4.58-fold of control. However, in cells treated with ACN, the CYP1 enzymatic activity was 0.27-fold of control, while the combination ACN-DOX showed no difference in activity against the control. Thus, ACN treatment alone reduced CYP activity compared to the control, and in the ACN-DOX combination, it inhibited the CYP induction caused by DOX (Figure 3, left panel). 

When compared to DOX-treated cells, the CYP1 enzymatic activities of ACN- and ACN-DOX-treated cells were 0.22- and 0.20-fold of DOX, respectively, further showcasing the effect of ACN when treating cells with DOX (Figure 3, right panel). Previous studies have reported the inhibition of CYP as an enhancer of drug efficacy. For instance, male rats orally treated with the flavonoid genistein showed an improved exposure of the chemotherapeutic drug paclitaxel when compared to untreated controls. Although not evaluated, the authors attributed this to the well-documented inhibitory effect of genistein on CYP enzymes [42]. In another study, female rats were co-administered quercetin orally with the chemotherapeutic drug Tamoxifen. Results also showed a higher concentration of the parental drug in plasma and lower metabolite concentration. These authors also attributed their results to the inhibitory effect that quercetin has on CYPs, specifically CYP3A4. Interestingly, the authors also highlighted that quercetin’s inhibition of P-gp could have led to the higher bioavailability of Tamoxifen in the quercetin-treated group compared to the control group [43]. A study that proved this theory administered carnosol along with cisplatin in MCF-7 tumor-bearing mice. Although no route of administration is specified, the results showed that carnosol significantly decreased CYP1A1 levels in tumors. These results showed that the inhibition of CYP1A1 by carnosol led to apoptosis in breast cancer cells [44].

### 3.3. CYP Modulation by ACN Enhanced Cell Viability Inhibition by DOX

**Figure 4:**
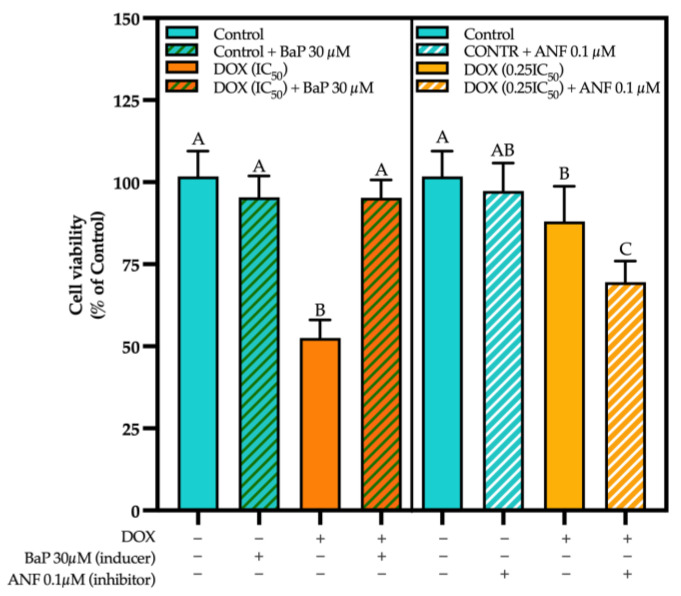
Cell viability is impacted by induction/inhibition of CYPs. Cell viability using a CYP inducer (left panel). Cells were treated with DOX (0–20 µg/mL) in presence or absence of CYP inducer Benzo[a]pyrene (30 µM). Cell viability using a CYP inhibitor (right panel). Cells were treated with DOX (0–20 µg/mL) in presence or absence of CYP inhibitor Alpha-naphthoflavone (1.5 µM). Cell viability and net growth (% of DMSO control) were determined as outlined in Section 2. Different capital letters indicate statistically significant differences (*p* < 0.05).

To evaluate whether CYP modulation contributes to the synergistic effect of ACN-DOX, the CYP inhibitor ANF and the CYP inducer BaP were utilized to test for cell viability. Results showed that inducing CYP activity with BaP enhanced cell growth in cells treated with DOX (95.23% ± 5.42 of control) at a dose that, when used alone, reduced cell viability to 52.52% ± 5.51 of control (DOX IC_50_) (Figure 4, left panel). 

In contrast, CYP inhibitor ANF significantly decreased cell viability in cells treated with DOX at 0.25×IC_50_ or the dose used in the combination ACN-DOX (69.54% ± 6.44 of control) vs. 88% ± 10.71 of control in cells treated with DOX (0.25×IC_50_ alone) (Figure 4, right panel). Therefore, the enhanced growth inhibitory effect of the ACN-DOX combination compared to DOX alone may be mediated by the ACN-inhibitory effect of CYP enzymatic activity. Contrarily, the induction of CYP activity made the drug less effective in inhibiting cell viability. Therefore, it can be concluded that reduced CYP activity decreases DOX metabolism, thereby enhancing its anticancer effects. Conversely, increased drug metabolism reduces its efficacy, as evidenced by cell viability results. This is supported by the well documented DOX metabolism mechanisms where, after being metabolized, DOX generates a secondary metabolite called Doxorubicinol which has shown less effectiveness in inhibiting cell growth [2]. These results are supported by studies demonstrating that CYP activity was significantly induced by DOX treatment. For instance, a study that exposed MCF-7 and 4T1 BC cells to tetrahydrocurcumin reported that its cell inhibitory effect was weakened when CYP1A1 was overexpressed. This study also mentioned that this overexpression led to increased survival in cancer cells [13], which resembles the results in this study. Another study reported that CYP enzymatic activity was inhibited in human breast cancer MCF-7 cells treated with the polyphenolic curcumin in a dose-dependent manner [45]. Similar results on the decrease of CYP activity were found when exposing the same cell line to the flavones Baicalein and Resveratrol [46,47].

Furthermore, it is well known that both CYPs and ABC transporters are targets for overcoming DR. Therefore, the efflux inhibition of anti-cancer drugs by phase III transporters was also studied.

### 3.4. ACN Target Phase III Drug Transporters

**Figure 5:**
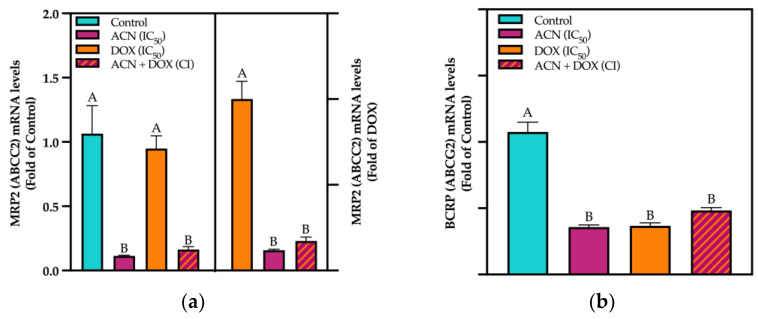
ACN inhibited ABC transporters in TNBC 4T1 cells at gene expression levels. (**a**) ABCC2 and (**b**) ABCG2 mRNA levels. Cells were treated with DMSO, ACN (IC_50_), DOX (IC_50_), and ACN-DOX (CI) for 6 h. mRNA analysis was performed as outlined in Section 2. Values are average (*n* = 3) ± SEM, *p* < 0.05 compared to DMSO controls. Different capital letters indicate statistically significant differences (*p* < 0.05).

Phase III drug transporters are membrane proteins that actively transport a wide range of substrates across cellular membranes. Among the phase III drug transporters that play a critical role in the pharmacokinetics and pharmacodynamics of drugs used to treat BC are BCRP (ABCG2), MRP2 (ABCC2), and P-gp (MDR1/ABCB1) [48]. For ABCC2 gene expression (encoding for MRP2 protein), results showed that ACN and ACN-DOX downregulated the mRNA levels to 0.11- and 0.17-fold of control, respectively. While DOX showed no difference against the untreated control (Figure 5a, left panel).

Using DOX as a control, it was shown that both ACN-DOX and ACN downregulated ABCC2 mRNA levels to 0.17- and 0.11-fold of DOX, respectively (Figure 5a, right panel). As for MRP2 (ABCC2) protein levels, only ACN-DOX and DOX showed a downregulation against control (Supplementary Figure S1).

The BCRP (encoded by the ABCG2 gene) is a transporter associated with resistance to chemotherapeutic agents; elevated BCRP expression in BC can lead to poor drug accumulation and treatment failure. Results showed ABCG2 mRNA downregulation by ACN-DOX, DOX, and ACN down to 0.48-, 0.36-, and 0.35-fold of control, respectively (Figure 5b). BCRP protein encoded by ABCG2 gene showed no suppression by any treatment (Supplementary Figure S1). Although BCRP is a well-known marker for drug resistance in breast cancer cells, ABCG2 generally does not act as a substrate for DOX unless a mutation occurs [49]. This could explain the observed lack of difference between treatments at the gene expression level and the lack of response at the protein level.

**Figure 6:**
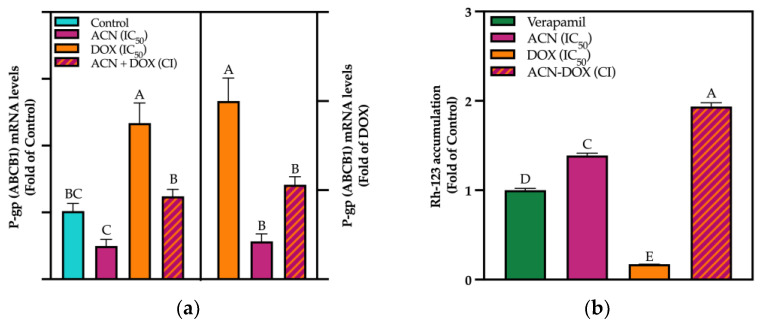
ACN reduced P-gp mRNA levels and allowed for more drug accumulation. (**a**) ABCB1 mRNA levels. Cells were treated with DMSO, ACN (IC_50_), DOX (IC_50_), and ACN-DOX (CI) for 6 h. mRNA analysis was performed as outlined in Section 2. Values are average (*n* = 3) ± SEM, *p* < 0.05 compared to DMSO controls. (**b**) Effect of treatments in P-gp activity. P-gp activity was assessed by accumulation of Rhodamine-123 (Rh-123) on the cell. Effect of treatments was compared to a positive control of a known P-gp inhibitor (Verapamil at 25 µM). After treatment, cells were exposed to Rh-123 to track P-gp activity by quantifying Rh-123 efflux outside of the cell. Accumulation was assessed by reading supernatant RFU and comparing it with initial concentration. Results are presented as mean (*n* ≥ 6) ± SD. Different capital letters indicate statistically significant differences (*p* < 0.05).

P-gp is a member of the ATP-Binding Cassette (ABC) transporter family and uses energy derived from ATP hydrolysis to pump substrates out of cells. It is particularly important in DR as it expels chemotherapeutic agents from cancer cells, reducing their intracellular concentrations and effectiveness. Results from mRNA levels of ABCB1 (responsible for encoding P-gp) showed that ACN downregulated its expression down to 0.49-fold of control, while DOX upregulated to 2.33-fold of control, and in the combination ACN-DOX, the mRNA levels were like the control (Figure 6a, left panel). 

Moreover, when compared to DOX, the mRNA levels of ACN-treated cells were 0.21-fold of DOX and the addition of ACN to DOX as in ACN-DOX treatment suppressed ABCB1 gene expression down to 0.53-fold of DOX-treated cells (Figure 6a, right panel). The P-gp gene is frequently overregulated in various cancer types, including BC [50]. More specifically, it has been reported that DOX cannot reach the nucleus of 4T1 BC cells from metastatic tumors because of increased nuclear expression of P-gp. Interestingly, at the protein level, P-gp expression was significantly downregulated by ACN, DOX, and ACN-DOX (Supplementary Figure S1).

Variations in protein expression under these treatments may be attributed to the influence of chemotherapy drugs on the ubiquitin–proteasome system (UPS) and autophagy, both of which are activated by oxidative stress. These processes are involved in protein degradation, particularly those proteins involved in signaling pathways that regulate ABC genes, such as the PI3K/AKT/mTOR and MAPK pathways [51]. Additionally, a study found that oxidative stress decreases P-gp expression at the protein level, leading to its internalization to an intracellular location [52]. Therefore, the lower P-gp band intensity could be attributed to these reasons, indicating that protein expression analysis was not reliable enough to show the effect on P-gp. Consequently, the evaluation of drug efflux is more relevant in assessing the role of P-gp in the synergistic effect of the ACN-DOX combination.

The drug efflux mediated by P-gp showed that the Rh123 accumulation in 4T1 cells increased by 1.38-fold of VER (a positive control known to inhibit P-gp) in ACN-treated cells. The DOX treatment suppressed Rh123 cell accumulation down to 0.17-fold of VER, while ACN-DOX treatment increased Rh-123 cell accumulation to 1.93-fold of VER and 11.35-fold of DOX (Figure 6b). In line with these findings, a prior investigation indicated that green tea polyphenols hindered the binding of drugs to these transporters, specifically P-gp, as well as the expulsion of drugs in Caco-2 human intestinal adenocarcinoma cells [6]. Similarly, a research study using human cervical epithelioid carcinoma (HeLaS3 cells) and a multidrug resistant human cervical cancer cell line demonstrated the inhibitory effect of caffeic acid in P-gp [20]. Interestingly, the better absorption of DOX orally in rats was attributed to the inhibition of P-gp coupled with the inhibition of a CYP protein by myricetin [53]. Although these results are promising, examining Rh-123 efflux alone does not clarify the mechanism by which ACN interacts with ABCB1, nor whether it reduces drug efflux or involves a reduction in drug uptake. Therefore, additional assays investigating ABC transporter activities should be included to provide a broader perspective on these transporters and their interaction with ACN. Nonetheless, given the crucial role of P-gp in drug resistance, further evaluation of its contribution to the ACN-DOX synergism was conducted.

### 3.5. ACN-Induced Drug Efflux Inhibition Contributed to the ACN-DOX Synergism

**Figure 7:**
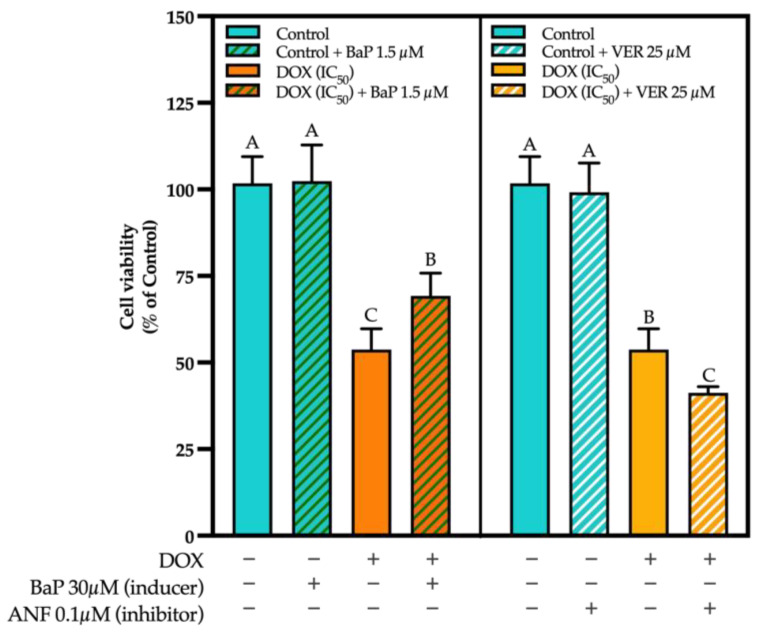
P-gp inhibition/induction showed influence in cell viability in TNBC 4T1 cells. Effect of P-gp inducer BaP and P-gp inhibitor Verapamil (VER) on cell viability. Cells were treated with DOX (0–20 µg/mL) in presence or absence of P-gp inducer BaP (1.5 µM) or P-gp inhibitor VER (25 µM). Cell viability and net growth (% of DMSO control) were determined as outlined in Section 2. A one-way ANOVA followed with Tukey’s multiple comparisons test was performed in all tests using GraphPad Prism. Different capital letters indicate statistically significant differences (*p* < 0.05).

To further demonstrate that P-gp inhibition contributes to the synergistic effect of ACN-DOX, cells were treated with the P-gp inducer BaP or the P-gp inhibitor VER in the presence of DOX. The results showed that inducing P-gp significantly increased cell viability in cells treated with the DOX IC50 dose compared to the treatment without an inducer (69.23 ± 6.57% of control and 53.72 ± 6.03% of control, respectively). Likewise, suppressing P-gp with VER enhanced significantly the cell viability inhibitory activity of DOX alone (41.15 ± 1.81% of control vs 53.72 ± 6.03% of control when using DOX alone) as shown in Figure 7. These results further confirm the well-documented fact that increased P-gp activity lowers the effects of chemotherapeutic drugs. However, when treated with ACN, this can be reversed by reducing P-gp activity, thereby preventing the drug from being pumped out of the cell.

These results strongly suggest that these two mechanisms, CYP inhibition and P-gp inhibition, may be the underlying mechanisms by which a synergism was found in ACN-DOX when in combination with a low dose of DOX (0.25× IC50).

This suggests that incorporating ACNs, either as extracts from DSCs or directly from the fruit, into a regular diet may enhance drug effectiveness without requiring dose escalation to levels that could cause harmful side effects to normal cells. Overall, the findings from this study indicate that ACN holds potential in preventing and/or reducing drug resistance by inhibiting DOX metabolism and DOX efflux from cells.

## 4. Conclusions

The co-administration of DOX with other molecules is promising for reducing its side effects. Polyphenols have shown potential for combination with DOX due to their role in overcoming drug resistance. Their effects on drug-metabolizing enzymes and drug efflux pumps have been reported [9,36]. In this study, cell treatment with ACN (at IC_50_ dose) and DOX (at 0.25× IC_50_) exerted synergism in inhibiting cell viability (CI = 0.6353). The increased cytotoxicity of ACN-DOX, even using a lower DOX dose (0.25× IC_50_), compared to DOX alone (IC_50_ dose), was in part attributed, to the ACN-mediated inhibition of P-gp drug efflux and CYP enzymatic activity. While numerous studies have explored the use of polyphenols to address drug resistance, there is limited research on their combined effects with DOX. Most studies primarily focus on how polyphenols modulate P-gp activity, which is well-documented [6,20,53,54,55]. Given that previous studies have suggested a potential relationship between the effects of polyphenols on P-gp and CYPs, it is crucial for future research to investigate both mechanisms, an approach which is also scarce in the literature.

The ability of ACN to inhibit P-gp drug efflux and CYP enzymatic activity highlights its potential to combat drug resistance when included in the diet, thereby enhancing drug efficacy. Therefore, the implementation of ACN into a BC patient’s diet should be studied to address its impact in drug efficiency.

Further studies are required to evaluate the effect of ACNs on enhanced drug toxicity and validate their role on CYP1s and the ABC phase III transporters. Recommended studies to further investigate these mechanisms include a guaranteed follow-up study with 4T1 drug-resistant cells. Additionally, an in vivo study could further validate these results and offer guidance into which administration routes might be more effective for implementing these treatments. It is important to note that results in this study shows the effect of ACNs on P-gp and CYP activity in 4T1 TNBC cells, but their mechanisms could be better explained in cell lines that show an overexpression of P-gp after acquiring drug resistance. This study is limited to a single cell line; therefore, research involving different TNBC cell lines could provide insight into the feasibility of these mechanisms.

## Figures and Tables

**Table 1:** ACN and DOX dose combinations used for synergistic study.

**ACN (IC_50_)**	**DOX (IC_50_**)			
	0.25×	0.5×	1×	1.5×
0.25×	0.25:0.25	0.25:0.5	0.25:1	0.25:1.5
0.5×	0.5:0.25	0.5:0.5	0.5:1	0.5:1.5
1×	1:0.25	1:0.5	1:1	1:1.5
1.5×	1.5:0.25	1.5:0.5	1.5:1	1.5:1.5

**Table 2:** Primer sequences.

Primer	Sequence
CYP1A1	Forward 5′-GGAACTAGACACAGTGATTG-3′
	Reverse 3′-TTGGGGATATAGAAGCCATTC-5′
CYP1B1	Forward 5′-ACTATTACGGACATCTTCGG-3′
	Reverse 3′-ATCTGGTAAAGAGGATGAGC-5′
ABCB1	Forward 5′-CCCATCATTGCAATAGCAGG-3′
	Reverse 3′-GTTCAAACTTCTGCTCCTGA-5′
ABCC1	Forward 5′-GAGTCAAAGCCGGTGGAAAAT-3′
	Reverse 3′-TTAGCTCCAGCCTTCTGCAGTT-5′
ABCG2	Forward 5′-AGCTCCGATGGATTGCCAG-3′
	Reverse 3′-GAGGGTTCCCGAGCAAGT-TT-5′
RPL-19	Forward 5′-GAAGGTCAAAGGGAATGTGTTC-3′
	Reverse 3′-CCTTGTCTGCCTTCAGCTTGT-5′

**Table 3:** Cell viability (% of control) of ACN and DOX treatments in 4T1 TNBC cells.

ACN (µg C3R/mL)	Cell Viability (% of Control) (Mean ± SD)
0	100
74.12	93.81 ± 7.04
148.24	84.21 ± 7.88
296.49	18.30 ± 5.07
**DOX** **(µg/mL)**	**Cell Viability (% of Control)** **(Mean ± SD)**
0	100
0.655	73.10 ± 8.02
1.25	66.75 ± 10.28
2.6	47.89 ± 7.65
3.93	36.49 ± 11.19

**Table 4:** CI values of ACN and DOX combinations ^1^.

ACN (IC_50_)	DOX (IC_50_)	Combination Index (CI)
0.25×	0.25×	1.3064
0.25×	0.50×	1.3145
0.25×	1×	2.9162
0.25×	1.5×	4.0968
0.50×	0.25×	0.9102
0.50×	0.50×	1.2126
0.50×	1×	1.8487
0.50×	1.5×	2.3838
1×	0.25×	0.6353
1×	0.50×	0.6908
1×	1×	0.8071
1×	1.5×	1.0344
1.5×	0.25×	0.8602
1.5×	0.50×	0.9208
1.5×	1×	1.0409
1.5×	1.5×	1.1391

^1^ Cells were treated with different proportions of ACN IC5_0_ and DOX IC_50_. CI values were calculated using CompuSyn software (www.combosyn.com, accessed on 12 June 2023). CI analysis of ACN and DOX effect determined as synergism for values 0.30 ≤ CI < 0.70, moderate synergism for values 0.70 ≤ CI < 0.85, slight synergism for values 0.85 ≤ CI < 1, additive for CI = 1, and antagonist for CI > 1, as determined by the program’s user’s guide.

## Data Availability

The raw data supporting the conclusions of this article will be made available by the authors on request.

## Supplementary Materials

The following supporting information can be downloaded at: https://www.mdpi.com/article/10.3390/cimb47030213/s1.

## Abbreviations

The following abbreviations are used in this manuscript:

DSC	dark sweet cherries
ACNs	anthocyanins
BC	breast cancer
TNBC	triple negative breast cancer
DR	drug resistance
DOX	Doxorubicin
CYP	Cytochrome P450
P-gp	P-glycoprotein
HER2	human epidermal growth factor receptor type 2
ER	estrogen receptor
PR	progesterone receptor
AhR	aryl hydrocarbon receptor
ABC	ATP-Binding Cassette
MDR-1	multidrug resistance protein 1
ABCB1	ATP-Binding Cassette Subfamily Member 1
BCRP	BC resistance protein
ABCG2	ATP-Binding Cassette Subfamily G Member 2
MRP2	multi-drug resistance protein 2
ABCC2	ATP-Binding Cassette Subfamily C 2
CI	combination index
ANF	alpha-naphthoflavone
BaP	Benzo[a]Pyrene
Rho	Rhodamine-123
VER	Verapamil
