# Supplementary material for: Dark Sweet Cherry (Prunus avium L.) Juice Phenolics Rich in Anthocyanins Exhibit Potential to Inhibit Drug Resistance Mechanisms in 4T1 Breast Cancer Cells via the Drug Metabolism Pathway
Source: Curr Issues Mol Biol. 2025 Mar 20;47(3):213. doi: 10.3390/cimb47030213 (PMC11941269; doi:10.3390/cimb47030213)
Supplement: Supplementary file 1 [file cimb-47-00213-s001.zip › cimb-3519182-supplementary.pdf]

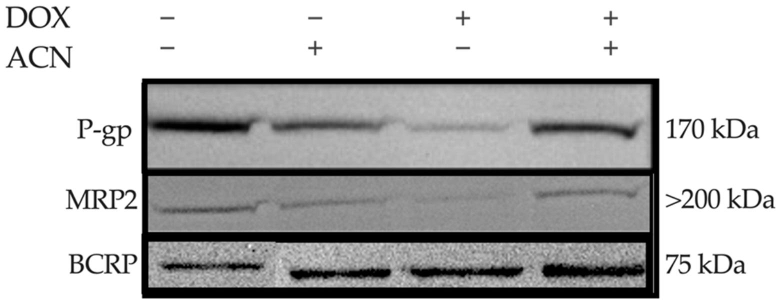

Figure S1. Effect of ACN, DOX, and ACN-DOX on expression of P-gp, MRP2 and BCRP proteins. Cell lysates were obtained after 24 h treatment with DMSO or ACN (IC_{50}), DOX (IC_{50}), and ACN-DOX (CI) and subjected to Western blot analysis using ß-actin as a loading control.
